# Properties of Guar Gum/Pullulan/Loquat Leaf Extract Green Composite Packaging in Enhancing the Preservation of Chinese Water Chestnut Fresh-Cut Fruit

**DOI:** 10.3390/foods13203295

**Published:** 2024-10-17

**Authors:** Kok Bing Tan, Meixia Zheng, Junyan Lin, Yujing Zhu, Guowu Zhan, Jianfu Chen

**Affiliations:** 1 College of Chemical Engineering, Academy of Advanced Carbon Conversion Technology, Huaqiao University, 668 Jimei Avenue, Xiamen 361021, China; 2 Agricultural Bio-Resources Research Institute, Fujian Academy of Agricultural Sciences, Fuzhou 350003, China; 3 College of Food Engineering, Zhangzhou Institute of Technology, Zhangzhou 363000, China

**Keywords:** guar gum/pullulan, loquat leaf extract, green packaging, Chinese water chestnut

## Abstract

Loquat leaf extract (LLE) was added to guar gum and pullulan as an environmentally friendly packaging film (GPE) to preserve Chinese water chestnuts (CWCs). The effect of the amount of LLE on the guar gum/pullulan composite film was investigated. The optimal amount of LLE was 4% (GPE4), with lower water vapor permeability (WVP) and greater mechanical strength, antioxidant, and comparable antibacterial performance than many pullulan-based films. Upon packing the CWCs for 4 days, the weight loss rate of GPE4 was only 1.80 ± 0.05%. For GPE4, the POD activity, the soluble solid content, and the vitamin C (Vc) content of the CWCs were 21.61%, 36.16%, and 26.22% higher than those of the control sample, respectively. More importantly, GPE4 was effective in preserving the quality of CWCs after 4 days of storage, better or at least comparable to non-biodegradable plastic wrapping (PE). Therefore, it can be concluded that GPE films hold significant promise as a sustainable alternative packaging material for preserving fruit-based foods like CWCs, potentially replacing PE in the future.

## 1. Introduction

Fresh fruits, such as *Eleocharis tuberosa* or Chinese water chestnut (CWCs), [1] are loved by consumers due to their freshness and convenience during consumption [2]. However, fresh-cut CWCs suffer serious mechanical injuries due to peeling and quality problems, such as enzyme browning (yellowing) and spoilage, that easily occur during shelf life, affecting edible quality and greatly reducing commercial value [3]. Therefore, an excellent packaging material for freshly cut CWCs is essential to preserve its quality. Currently, plastic packaging is widely used in daily life for food preservation [4], including CWCs. However, it is non-biodegradable and very harmful to the environment upon disposal [5]. Therefore, people have begun to actively study the technology of producing films using green natural extracts and biodegradable polymer materials to promote the green transformation of the food packaging industry and ensure food safety.

Pullulan is a type of natural water-soluble biomaterial synthesized via fermentation with *Aureobasidium* pullulan [6]. It is non-toxic, has a good oxygen barrier [7], and has antibacterial abilities [8]. By utilizing the unique ability in linkage, it has an excellent film formation property, allowing it to be widely used in food preservation applications [6,9]. However, the pure pullulan film suffers from weak mechanical properties with limited antioxidant properties [10].

To overcome these weaknesses, pullulan is usually composited with other materials [11,12,13,14,15,16,17]. Guar gum is a natural polysaccharide compound with galactomannan as the main component [18]. It has the advantages of safety, non-toxicity, good biocompatibility, and complete biodegradation, making it a suitable alternative to conventional non-biodegradable plastic packaging material [19]. Although guar gum has antioxidant properties [20] and can improve the mechanical strength of the polymeric film [21], it is prone to bacterial attacks, inhibiting its application as a packaging material [22]. As mentioned above, pullulan has good antibacterial properties, but weak antioxidant properties. Therefore, pullulan and guar gum will complement each other’s strengths and weaknesses in terms of mechanical strength and antioxidant and antibacterial performance, which are essential properties of food packaging. However, both guar gum and pullulan are known for their high WVP [23,24,25,26]. High WVP will promote the volatilization of nutrients during storage due to the transpiration of water [27]. This inhibits the practicality of the packaged materials developed for potential commercialization. Therefore, both the gum guar and pullulan films are usually composited with other materials to decrease WVP [23,24,25,26].

Loquat leaf extract (LLE) is known to be rich in highly active ingredients, such as ursolic acid [28]. The pentacyclic structure of ursolic acid provides many interesting properties of LLE such as antibacterial [29] hydrophobicity [30] and antioxidant [31]. Therefore, LLE has been traditionally used for the treatment of chronic bronchitis, cough, phlegm, and high fever due to bacterial infection [32], while it can also be used to decrease WVP and improve the antioxidant property of the biomaterial film, such as ripe banana peel/starch [27]. Furthermore, LLE also contains antioxidants, such as triterpenic acid [33], flavonoids [34], and polyphenols [35]. Therefore, by compositing LLE in pullulan/guar gum, it is hypothesized that LLE will play a role as a bioactive component in improving the antibacterial and antioxidant properties of the film. More importantly, using the hydrophobicity property of ursolic acid in LLE, it is also hypothesized that a pullulan/guar gum-based film with high mechanical strength yet low WVP can be developed. Hence, it is hypothesized that a practical packaging material can be developed to preserve the quality of fresh-cut fruit, such as the CWC for potential commercialization. However, to the authors’ knowledge, there are no studies or limited studies on the film synthesis of guar gum/pullulan with LLE.

Therefore, in this study, the composite film was prepared using guar gum and pullulan as the substrate, glycerol as the plasticizer, and LLE as the bioactive component to develop a practical bio-based packaging material with excellent antibacterial, antioxidant, and mechanical properties with low WVP to preserve fresh-cut CWCs. The effect of the amount of LLE on the structure, WVP, light transmittance, antioxidant, and antibacterial performance of the composite film was investigated. More importantly, the effect of LLE on the guar gum/pullulan composite on food packaging and the preservation of CWC fresh-cut fruit was investigated.

## 2. Materials and Methods

### 2.1. Materials

Fresh CWCs were purchased at a supermarket in Zhangzhou without mechanical damage, pests or diseases, and consistent maturity. Full fruits were selected as experimental materials. Food-grade guar gum, was purchased from Guangdong Zhenweiyuan Food Ingredients Co., Ltd., Guangdong, China. Food-grade pullulan, was purchased from Henan Junyi Biotechnology Co., Ltd., Henan, China. Similarly, LLE (30: 1 extract, food grade) was purchased from Shaanxi Haiyisi Biotechnology Co., Ltd., Shaanxi, China, and food-grade glycerol was purchased from Aladdin Industries. In addition, all other chemical reagents used are analytical grade.

### 2.2. Methods

#### 2.2.1. Preparation of Guar Gum/Pullulan/LLE Composite Film (GPE)

A suitable amount of guar gum was weighed, soaked with 95% ethanol, and then prepared into a 2% solution with deionized water. Similarly, a proper amount of pullulan was weighed and prepared into a 1% solution with deionized water. Subsequently, 45 mL of guar gum solution and 0.20 g of glycerol were evenly mixed. Next, 0%, 2%, 4%, and 6% LLE powder were added (weight percent with respect to the amount of guar gum), and the mixture was stirred for 10 min until the LLE powder was completely dissolved. Then, ultrasonic was used for defoaming. The resulting solution was poured into a Petri dish, cast, dried to form a film, and stored in a dryer for later use. The films prepared to contain 0%, 2%, 4%, and 6% loquat leaf powder were labeled GPE0 (0%), GPE2 (2%), GPE4 (4%), and GPE6 (6%), respectively, to facilitate identification and differentiation in subsequent experiments or applications.

#### 2.2.2. Thickness of the GPE Film

A flat-headed thousandth thickness gauge (Awt-chy01, Henan Bangte Gauge Co., Ltd., Henan, China) was adopted to measure the thickness of the film according to GB/T 1040.3-2006 according to our previous study [36], with slight modification. Nine different test points were selected on the diagonal of the film sample at equal intervals, whereby the average thickness was measured in micrometers. All of these steps were based on Chen et al. [36].

#### 2.2.3. Light Transmittance of GPE Film

The composite film was shaped into a 6 mm diameter disc via a puncher and then placed on the plate. It was then scanned with an enzyme-labeled instrument (MultiSkan Go, Thermo Fisher Scientific Shier Technology Co., Ltd., Waltham, MA, USA) in the wavelength range of 200~800 nm.

#### 2.2.4. Water Vapor Permeability (WVP) of the GPE Film

The WVP of the composite film was measured using the cup test method according to GB/T 1037-1988 [37] with slight modification. Specifically, anhydrous CaCl_2_ was dried at a constant weight, and a particle diameter of less than 2 mm was selected and placed in a weighing bottle until it was 5 mm from the mouth of the bottle. The composite film was then covered on the bottle’s mouth, and a rubber band was used to seal it to ensure good air tightness. Then, the bottle was weighed, and its initial weight was recorded. Subsequently, the weighed weighing bottle was placed in a biochemical incubator and kept at 25 °C and 100% relative humidity for 48 h [37]. After that, it was taken out and weighed again, and this process was repeated three times. Finally, the WVP was calculated according to the following method [5]:

*WVP* = (*m* × *L*)/(*A* × *t* × *ΔP*) (1)

In the formula, *m* is the mass (g) of water that penetrates the film [5]; *L* is the thickness (m) of the composite film [5]; *A* is the effective area (m^2^) of the composite film [5]; *t* is the passage time (s) of moisture; and *ΔP* is the difference in vapor pressure (Pa) between the two sides of the composite film.

#### 2.2.5. Water Solubility of GPE Film

The composite film was cut to specifications of 30 mm × 30 mm and then dried in an oven at 105 °C to a constant weight of *m*_0_ (g). Then distilled water was added and the composite film was soaked for 24 h at room temperature. After filtration, the insoluble composite film was obtained, which was dried in an oven at 105 °C again and until a constant weight *m_t_* (g) was reached. The experiment was repeated three times. The *WS* calculation formula is as follows [5]:

*WS*/% = (*m*_0_ − *m_t_*)/*m*_0_ × 100% (2)

#### 2.2.6. Moisture Content (MC) of GPE Film

With 30 mm × 30 mm specifications, the composite film was exposed to room temperature for 48 h to obtain the mass *m*_0_ (g). The composite film was then inserted into an oven at 105 °C and dried until the constant weight of *m_i_* (g). The experiment was carried out in parallel three times, and the *MC* calculation formula is as follows [5]:

*MC* = (*m*_0_ − *m_i_*)/*m_i_* × 100% (3)

#### 2.2.7. Mechanical Properties of GPE Film

The film’s mechanical properties were measured to GB/T 13022-1991 (Chinese National Standard) testing method as conducted in our previous study [36] but with slight modification. First, the composite film was cut into 4 mm × 20 mm × 20 mm strips, and the mechanical properties were tested using a texture analyzer (CT3-10K, Bolefei Company, Canton, MS, USA). During the testing process, the composite film was stretched at a speed of 1 mm/s, and its initial length *L*_0_, the maximum length *L*, and the maximum pulling force *F* (N) were recorded at the time of fracture. The experiment was carried out in parallel three times, and the tensile strength (*TS*) and elongation at break (*EAB*) were obtained [38].

*TS* (MPa) = *F*/(*b* × *d*) (4)

Here, *b* represents the width of the film (mm) and *d* represents the thickness of the film (mm).

*EAB* = (*L* – *L*_0_)/*L*_0_ × 100% (5)

#### 2.2.8. Fourier Transmission Infrared (FTIR) of a GPE Film

The infrared spectrum of the sample was tested using a Fourier transmission infrared (FTIR) spectrum analyzer (NICOLET iS 10, Thermo Fisher Scientific Shier Technology Company). The ambient temperature was kept at 25 °C, and was scanned in the range of 4000~650 cm^−1^ wavelength, with 1 cm^−1^ step size 32 times.

#### 2.2.9. Crystallinity of GPE Film

The crystallinity of the film was measured via an Ultima IV X-ray diffractometer (XRD) at room temperature. The measurement was done with CuKα as the X-ray source 40 kV working voltage and 40 mA current. Meanwhile, the scanning range (2θ) was set at 5 to 80, 4 per minute scanning rate and 0.02 step size.

#### 2.2.10. Morphology of GPE Film

To explore the morphological characteristics of the composite film, the composite film was broken with liquid nitrogen and was fixed on the sample table for gold spraying to enhance its conductivity. Then the morphology of the composite film was observed by scanning electron microscopy (JSM-6010LA, JEOL) at an accelerated voltage of 20 kV.

#### 2.2.11. Antioxidant and Antibacterial Properties of GPE Film

For antioxidant measurement, the DPPH solution with a mass concentration of 0.10 g/L was prepared with 95% ethanol, and 2 mL of this solution was added with 2 mL of film solution. After shaking, it was placed in the dark at room temperature for 30 min and the absorbance *A_i_* was measured at 517 nm. At the same time, the absorbance *A*_0_ of 2 mL of 95% ethanol mixed with 2 mL of DPPH and the absorbance *A_j_* of 2.00 mL of film solution mixed with 2 mL of 95% ethanol were determined. The DPPH radical scavenging rate, which was expressed as *R*(%), was calculated as follows [5]:

*R*(%) = [1 − (*A_i_* − *A_j_*)/*A*_0_] × 100% (6)

Meanwhile, 250 mL ABTS^+^ solution with a concentration of 7 mmol/L and 250 mL K_2_S_2_O_8_ solution with a concentration of 2.45 mmol/L were prepared and mixed to be reacted in the dark at a temperature of 25 °C for a period of 12 h for the preparation of ABTS^+^ free radical reserve solution. The film solution was mixed with 2 mL of this solution, reacted in the dark for 10 min, and the absorbance was measured at 734 nm and recorded as *A_m_*. Next, 50% anhydrous ethanol of 2 mL was added film solution with a volume of 2 mL, and its light absorption value was measured and recorded as *A_n_*. Simultaneously, ABTS^+^ solution of 2 mL and 50% ethanol of 2 mL were mixed, and the resulting light absorption value was measured and recorded as *A_k_*. The scavenging rate of ABTS^+^ free radicals was expressed as *Q*(%) and its calculation method is as follows [39]:

*Q*(%) *=* [1 − (*A*_1_ − *A*_2_)/*A*_0_] × 100% (7)

The antibacterial activity of the composite film was determined using the inhibition zone method [40]. *Staphylococcus aureus* (ATCC6538), *Escherichia coli* (ATCC25922), and *Bacillus subtilis* (ATCC6633) were selected as test strains. First, these strains were inoculated into 10 mL of bacterial culture solution, evenly mixed, and cultured for 24 h on a shaking table at 37 °C at a speed of 120 rpm. The activated bacterial suspension was then diluted to a suitable dilution, and 0.1 mL of the diluted solution was sucked into the nutrient agar plate with a sterile straw and evenly smeared with a sterile coating stick. The composite film with a diameter of 6 mm was then placed in the solid culture medium coated with bacterial liquid and cultured at 37 °C for 24 h. Finally, the diameter of the bacteriostatic zone was measured using a Vernier caliper to evaluate the antibacterial performance of the composite film.

#### 2.2.12. Composite Film as Food Packaging for CWCs

To test and compare the preservation performance of CWCs using GPE film and plastic film (PE), the CWC was first cleaned with distilled water. It was then dried, peeled, and cut into 6 dice pieces of 1 cm × 1 cm × 1 cm. The dice pieces were soaked in 1% NaClO solution for 3 min and were then removed from the solution and rinsed with distilled water. One of the dice pieces without any wrapping was labeled as a “control” sample, while the remaining 5 dice pieces were then placed on a tray and were wrapped with 5 different wrappings, which were labeled as PE, GPE0, GPE2, GPE4, and GPE6 samples. The samples were stored at room temperature (25 °C) for 4 days. The samples were stored at room temperature (25 °C) for 4 days. The quality of the fresh-cut concerning appearance, weight loss rate, vitamin C (Vc) content, peroxidase (POD) activity, and soluble solid content of CWCs were evaluated at day 0 and day 4.

The weight loss rate of CWCs was measured using the weighing method, and the weight *W*_0_ of CWCs was recorded in different treatment groups in the initial preservation stage (day 0), while the weight *W_t_* of CWCs was on the last day (day 4). The weight loss rate (*WLR*) was calculated according to the following formula [5]:

*WLR* = (*W*_0_ − *W_t_*)/*W*_0_ × 100% (8)

The method of determining Vc is based on the 2,6-dichlorophenol indophenol titration method of GB 5009.86—2016 “Determination of Ascorbic Acid in the National Standard of Food Safety” [41]. Specifically, CWCs were measured in weight (*m*, g), and a small amount of 2% oxalic acid was added to grind it into homogenate. A 2% oxalic acid amount was inserted into a 100 mL volumetric flask to a constant volume and was shaken evenly. Upon filtration, 10 mL volume of filtrate was pumped into a 50 mL triangular flask, by which it was titrated pink with a calibrated 2,6-dichloroindophenol solution without fading for 15 s to obtain a volume *V* titration (mL). The calculation formula of the Vc content, *X* (mg/100 g) is as follows [41]:

*X* = [(*V* − *V*_0_) × *T* × *A*]/*m* × 100% (9)

where *T* is the titration of the T-2,6-dichloroindophenol solution, mg/mL and *A* is the amount of dilution times.

The CWC was ground and crushed, homogenized by high-speed centrifugation, and filtered by gauze, the soluble solid content of the juice was determined by the digital sugar meter PAL-1 of Aituo Sugar Meter in Japan. The measurement was repeated three times and the average value was taken. For the measurement of POD activity, 5 g of fresh peeled CWC was mixed with 5 mL of buffer (1 mmol/L PEG 6000, 40 g/L PVPP, and 10 g/L Tritonx-100). The homogenate was then manually ground and centrifuged at 12,000 r/min for 30 min, and the supernatant was collected. Subsequently, the unit of POD activity was defined as the amount of enzyme needed for the absorbance change of 0.01 per gram of fresh CWC at 470 nm/min, which was expressed as U g^−1^. These steps were performed for soluble solid content.

#### 2.2.13. Statistical Analysis

Data were processed using DPS v7.05 software and Origin 8.5 software, and the average standard error was obtained. Significant analysis was conducted based on samples developed in this study (control, PE, GPE0, GPE2, GPE4, and GPE6), where the Duncan multiple comparison method is selected and *p* < 0.05 is set as the significance level. The significance level is represented by the different lowercase letters a, b, c, and d, on each sample, relative to the results demonstrated by the subsequent sample listed in the figures and tables. In the figures and tables, if the lowercase letter of a sample is the same as the subsequent sample, then there is no significant difference in results between these two samples. However, if the lowercase letter of a sample is different from the subsequent sample, then there is a significant difference in results between these two samples.

## 3. Results and Discussion

### 3.1. Characterization of GPE Film

**Figure 1:**
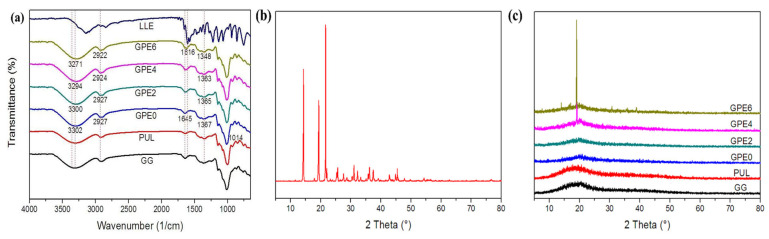
(**a**) FTIR of LLE, GG, pullulan, and composite films; (**b**) LLE XRD pattern of LLE; (**c**) LLE XRD pattern of composite film.

The FTIR spectra of LLE, guar gum (GG), and GPE films are shown in Figure 1a. The wide yet strong peak of the GPE0 film at around 3302 cm^−1^ is attributed to the vibration absorption peak of the O-H bond stretching [42]. Meanwhile, the two peaks at 2927 and 2895 cm^−1^ are attributed to the vibration absorption peaks of the alkyl C-H bond stretching [43]. Also, the absorption peak at 1645 cm^−1^ is attributed to the -O-C-O stretching vibration of the pullulan and guar gum in the composite film [44]. The absorption peak at 1014 cm^−1^ is the stretching vibration peak of the glycosidic bond C-O-C [45] of the composite film from guar gum [46] and pullulan [47]. After adding LLE, it was found that the GPE composite film had all of the characteristic peaks of GPE0. However, with an increasing LLE amount, the stretching vibration at 3302 cm^−1^ gradually shifted to 3271 cm^−1^ in the direction of the lower wavenumber. This indicated that the LLE could have interacted with the hydroxyl groups in the GPE0 film for the formation of hydrogen bonds [48]. A similar interaction was observed in the works of Gao et al. [49] and Chen et al. [5,36]. Similarly, the absorption peaks correspond to methylene C-H at 2927 cm^−1^, which shifted to 2922 cm^−1^ [43]. Furthermore, the absorption peak at 1616 cm^−1^ became more significant with a higher amount of LLE (GPE6), which is attributed to the C=O bond [50] from polyphenol [51], triterpenic acid [33] and ursolic acid [52] from LLE.

Figure 1b,c show the XRD patterns of loquat extract and composite film, respectively. As shown in Figure 1b, sharp peaks were observed in the XRD spectra for LLE, especially in the range of 2θ = 10~25, indicating that the LLE has a certain crystallinity and its grain size is large [53]. Figure 1c further reveals that a broad peak at about 2θ = 20.96 was observed on the GPE0 film, due to the amorphous structure of the GPE0 film. It should be noted that when 2% LLE was added to the film, the intensity of the diffraction peak was significantly enhanced and shifted to the right compared to the GPE0 film. This change revealed that there was a strong interaction between the LLE and the film. As a result, the entanglement between macromolecules was decreased. Consequently, the crystal structure of the film-forming polymer was changed, leading to an increase in the crystallization zone in the blended film. These changes in the XRD patterns fully show that LLE, guar gum, and pullulan have good biocompatibility. Furthermore, when the LLE content reached 4%, the crystallization peak of the LLE appeared at 2θ = 19.04, which may be due to unreacted excessive free LLE in the composite film system. Such results have been observed in the work of Martins et al. [54] and Hazirah et al. [38].

**Figure 2:**
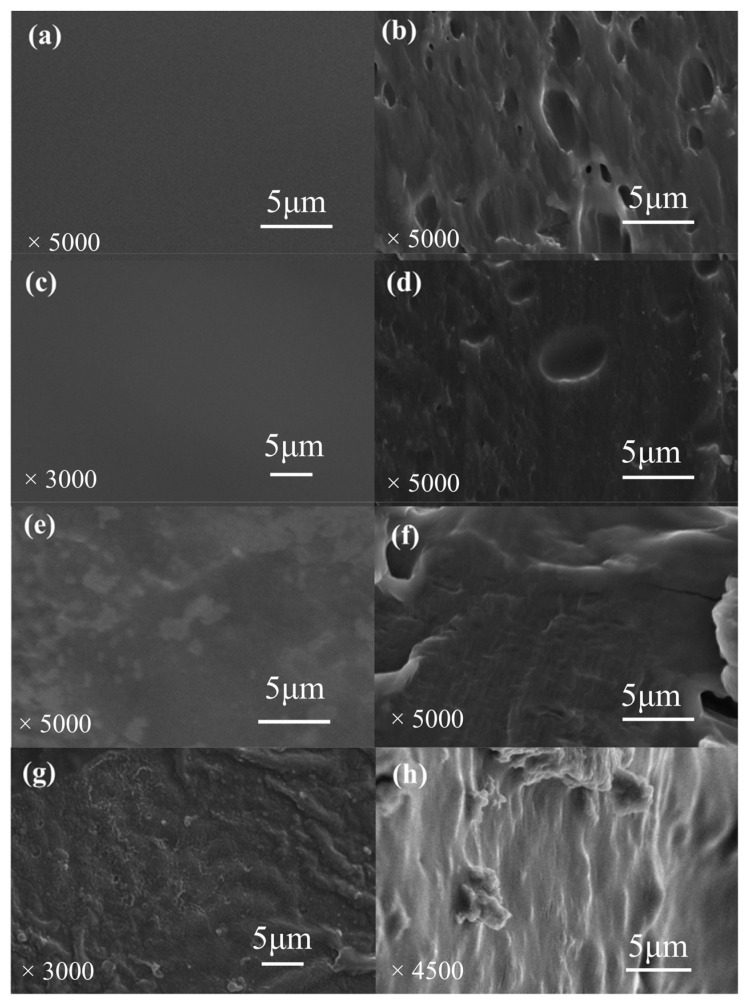
SEM images of (**a**) GPE0 surface; (**b**) GPE0 cross-section; (**c**) GPE2 surface; (**d**) GPE2 cross-section; (**e**) GPE4 surface; (**f**) GPE4 cross-section; (**g**) GPE6 surface; (**h**) GPE6 cross-section.

The SEM images of the surface and cross-section of the composite film are shown in Figure 2. It was observed that the surface morphology of the GPE0 film (Figure 2a) is rather smooth and flat. Meanwhile, in the cross-section of the composite film, the internal structure of GPE0 is relatively loose, and some unplanned pores are distributed in its cross-section (Figure 2b). When the amount of LLE added was low (GPE2) (Figure 2c), the surface remained smooth as the GPE0 film (Figure 2a). However, the cross-section (Figure 2d) became more compact than GPE0 (Figure 2b), whereby the pores of the cross-section structure of the composite film gradually became smaller and disappeared. Likewise, the hydrogen bonds also occurred between sodium alginate, tea leaf extract, and gelatin, resulting in the formation of compact film, as explained in the work of Shan et al. [55]. However, when the amount of LLE was 4% and above (Figure 2e,g), the surface of the composite film began to become rough, and some small particles and even some strip-shaped wrinkles appeared, indicating that the compatibility of LLE with the guar gum and pullulan matrix had begun to deteriorate and local agglomeration had formed. It should be noted that even though the GPE4 surface was wrinkled (Figure 2e), the cross-sectional structure (Figure 2f) was still relatively compact. However, when 6% LLE (GPE6) was added (Figure 2h), the cross-sectional structure of the film showed a rough structure instead and part of the film was agglomerated, which may be due to the excessive interaction between LLE and the matrix. This can also be observed when the amount of xanthan gum in agar composite film is excessive [56]. This shows that the optimum amount of LLE is essential to ensure excellent interaction between LLE and the composite film matrix and be well distributed in the matrix.

### 3.2. Effect of LLE Amount on Film Thickness, Moisture Content (MC), and Water Solubility (WS) of GPE Film

Table 1 lists in detail the effect of LLE amount on the thickness of the composite film. When comparing the data, it can be observed that with increasing amounts of LLE, the thickness of the composite film increased compared to the control film, but this change was not significant (*p* > 0.05). Specifically, the composite film thickness was found to increase from 0.152 ± 0.005 mm to 0.157 ± 0.009 mm when the amount of LLE gradually increased from 0 to 6%. This was mainly due to the interaction between LLE and guar gum and pullulan via hydrogen bonds (as verified in Figure 1a), which enhance the structural compactness of the composite film, thus forming a thicker film.

Moisture content (MC) is the key factor that affects the mechanical properties, stability, and degradation of edible films. If the MC is high, the edible film will become too soft, which will affect its operability in practical application; however, if the MC is too low, the edible film will become too brittle and easy to break. Meanwhile, water solubility (WS) is an index for measuring the water resistance of polymer films, which affects the stability of films in different humidity environments and their potential applications. records in detail the specific effects of LLEs with various amounts on the MC and WS of the film composite. As can be seen from the data in the table, the initial MC and WS of the GPE0 film were 22.94 ± 1.67% and 60.89 ± 1.25%, respectively. When 2% LLE was added (GPE2), the MC and WS of the film composite began to significantly decrease (*p* < 0.05) to 20.60 ± 2.09% and 44.25 ± 1.38%, respectively. Based on Figure 1a, the film became more compact due to the hydrogen bond between LLE and guar gum/pullulan composite, as observed in Figure 2d. Such interactions effectively prevent the formation of left-over intermolecular hydrogen bonds between GPE0 and water molecules [5,57]. This decreases the composite film’s solubility and the molecule’s mobility in the matrix structure, affecting the solubility of the composite film [5,57]. As a result, the composite film’s water resistance was improved. Such results can also be observed in the pullulan/hydroxypropyl methylcellulose composite film [58] developed by Yan et al. With an additional increase in the content of LLE, especially when the content is 4% and above, the film composites’ MC and WS did not decrease significantly (*p* > 0.05). Based on Figure 1c, the amount of LLE at 4% and beyond was too excessive, causing some free LLE in the film composite to remain unreacted.

### 3.3. Effect of LLE Amount on Mechanical Properties, WVP, and Transmittance

**Figure 3:**
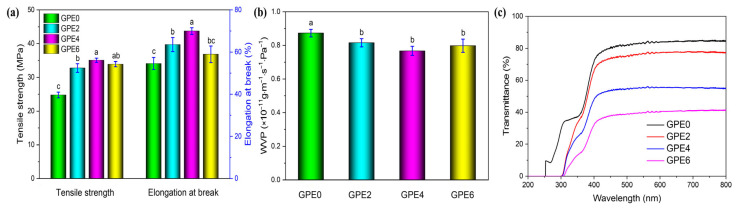
(**a**) Mechanical Strength; (**b**) WVP; and (**c**) Light transmittance of the composite film.

The food packaging film‘s mechanical properties are important, as it directly affects its durability in protecting the products during packaging, transportation, and storage [59]. Figure 3a clearly shows the effects of different LLEs on the mechanical properties of the composite film. The tensile strength of the composite film without LLE was found to be (24.9 ± 0.85) MPa, while the elongation at break is 84.6 ± 2.83%. With an increase in the LLE amount, the mechanical properties of the composite film showed a trend of first strengthening and then weakening. For example, when the amount of LLE was 4% (GPE4), the mechanical properties of the composite film reached the optimal state, where its tensile strength increased significantly to (35.08 ± 0.63) MPa, and its elongation at break also increased to 69.98 ± 1.58%, which were increased, respectively, by 41.51% and 28.17%, compared to the composite film GPE0, showing excellent tensile properties and properties. Based on Figure 2d,f, these interactions changed the original film structure and formed a more compact network structure, where the film’s mechanical strength was enhanced. At the same time, these active components in LLE can also have a “plasticising” effect on the matrix, improve the fluidity and mobility of molecules and polymer chains, and improve the elasticity of polymer chains. Hence, the film composite elongation at break was improved. However, it should be noted that when the amount of LLE was too large, especially at 6%, the interaction between molecules was inhibited by the unreacted free LLE, which consequently destroyed composite film network structure stability. This eventually led to a decrease in the film’s composite tensile strength and elongation at the break.

Figure 3b shows the effect of different amounts of LLE on the WVP of the composite film. The WVP of the composite film was found to decrease from (8.7277 ± 0.2246) × 10^−11^ g·m^−1^·s^−1^·Pa^−1^ (GPE0) to (7.6724 ± 0.2689) × 10^−11^ g·m^−1^·s^−1^·Pa^−1^ (GPE4). The results show that the addition of LLE can decrease the WVP of the composite film. As shown in Figure 1a, the hydrogen bond between LLE and guar gum/pullulan reduces the intermolecular gaps and promotes the formation of a closer network structure between film-forming substances [38]. Such a trend can also be observed in the work of Siripatrawan and Harte [60], Wang et al. [61], and Chen et al. of our group [62]. Furthermore, the ursolic acid component in LLE has hydrophobic properties [30]. These inhibited the diffusion of water vapor molecules and thus decreased WVP [31]. However, when the LLE content was at 6%, the WVP of the composite film began to show an upward trend. Nevertheless, its value was still lower than that of the GPE0 film without LLE.

**Figure 4:**
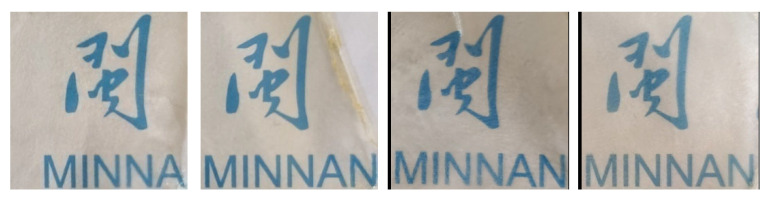
Physical diagram of the composite film.

Light transmittance is essential to assess the quality of packaged food’s appearance, which is directly related to the acceptance by consumers of packaged food. Figure 3c shows the influence of different contents of the LLE on the light transmittance of the composite film. It can be observed that the GPE0 film light transmittance fluctuates between 0 and 29.38% in the ultraviolet region (200~300 nm) and 37.07% to 84.72% in the visible region (350~800 nm) without adding LLE (Table 2). When the amount of LLE was increased, the transmittance of the composite film showed an obvious downward trend, which fully proved that LLE could effectively enhance the composite film’s performance in light inhibition, especially for ultraviolet light with a wavelength below 300 nm. When the LLE content reached 4%, the GPE4 film ultraviolet transmittance at 300 nm was only 0.011%. At the same time, with increasing LLE dosage, the color of the composite film turned yellow gradually due to LLE becoming yellowish [63]. Furthermore, the addition of LLE enhances the reflection, absorption, and refraction of light in the composite film, thus reducing the light transmittance. Nevertheless, each group of composite films can still clearly display the covered patterns (see Figure 4), proving that its light transmittance is still maintained at a good level. As a result, this composite film can effectively minimize or prevent food exposure to ultraviolet and visible light, hinder the light-induced breakdown of nutrients, and ultimately extend the shelf life of packaged foods.

### 3.4. Effect of LLE Amount on Antioxidant Activity and Antibacterial Properties

**Figure 5:**
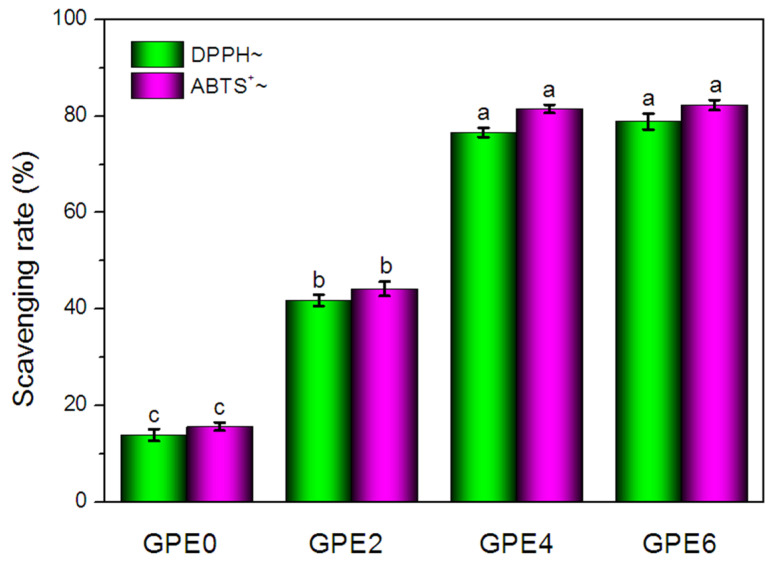
Antioxidant properties of composite film.

The composite film antioxidant capacity was evaluated based on DPPH and ABTS^+^ free radical scavenging methods [64]. As shown in Figure 5, the scavenging rates based on DPPH and ABTS^+^ of the GPE0 film were 13.87 ± 1.25% and 15.60 ± 0.82%, respectively. This demonstrated the weak free radical scavenging capacity, which may be due to the relatively weak antioxidant properties of guar gum and pullulan. However, after the introduction of LLE into the composite film, the scavenging ability of the composite film for DPPH and ABTS^+^ free radicals was significantly improved (*p* < 0.05). With the increasing concentration of LLE, the scavenging capacity of the composite film for DPPH and ABTS^+^ also showed a gradual and increasing trend. Specifically, when the LLE content reached 2% (GPE2), 4% (GPE4) and 6% (GPE6), the DPPH radical scavenging rate of the composite film was significantly greater to 41.79 ± 1.23%, 76.64 ± 0.94% and 78.88 ± 1.60%, which were 201.30%, 452.56% and 48.88%, higher than that of the GPE0 film, respectively. Meanwhile, the free radical scavenging rates of ABTS^+^ also increased to 44.19 ± 1.50%, 81.53 ± 0.84%, and 82.30 ± 1.04%, which were 183.27%, 422.63%, and 427.56% higher than that of the GPE0 film, respectively. The results showed that there was a dose-dependent relationship between the free radical scavenging rate of the composite film and the amount of LLE added. This is due to the compounds, such as triterpenic acid, ursolic acid, flavonoids, and polyphenols [28,34,35,65], contained in LLE, which endow LLE with excellent anti-free radical activity. However, it should be noted that when the amount of LLE was 6%, the increase in antioxidant activity was no longer significant (*p* > 0.05). The results showed that the addition of an optimal amount of LLE to the composite film was the key to achieving efficient antioxidant protection.

The antibacterial performance of the GPE film was evaluated by evaluating the antibacterial performance of the GPE film against the growth of *Escherichia coli*, *Staphylococcus aureus*, and *Bacillus subtilis*. The experimental results showed that the GPE film (GPE0) without LLE did not have an obvious antibacterial effect on the target bacteria. However, when LLEs (GPE2, GPE4, and GPE6) were added to the GPE film, their antibacterial properties against *Escherichia coli*, *Staphylococcus aureus*, and *Bacillus subtilis* improved significantly. Specifically, according to Table 3, as the amount of LLE increased from 2% to 6%, the diameters of the inhibition zones for *Escherichia coli*, *Staphylococcus aureus*, and *Bacillus subtilis* increased significantly from (7.82 ± 0.21) mm, (8.77 ± 0.17) mm and (7.12 ± 0.07) mm to (10.07 ± 0.09) mm, (10.96 ± 0.11) mm and (9.04 ± 0.09) mm, respectively. Further analysis of the data showed that the composite film had the most significant antibacterial effect on *Staphylococcus aureus*, followed by *Escherichia coli* and *Bacillus subtilis.*

Considering that *Bacillus subtilis spores* have strong resistance, the inhibition effect of the composite film on *Bacillus subtilis* is relatively weak [66]. Meanwhile, the antibacterial effect of the composite film on *Staphylococcus aureus* and *Escherichia coli* is different, mainly due to the difference in their cell wall structure. Unlike *Escherichia coli*, which has a complex cell wall structure, the cell wall structure of Gramme-positive bacteria, such as *Staphylococcus aureus*, is relatively simple, composed primarily of a layer of peptidoglycan and phosphomuramic acid, and does not contain a layer of lipopolysaccharide [36]. Therefore, the cell wall of *Staphylococcus aureus* is more susceptible to penetration and destruction by bacteriostatic agents.

However, when LLE was added to the film, the bacteriostatic effect on the composite film was improved, and the diameter of the bacteriostatic circle gradually increased with the increase in the amount of LLE. This is because the compounds, such as triterpenic acid, ursolic acid flavonoids, and polyphenols [28,34,35,65], contained in LLE can reduce the fluidity of the cell film or perforate the film through hydrogen peroxide, which then destroys the cell film of microorganisms and inhibits microbial growth [67]. Therefore, this study confirmed that the GPE film containing LLE has remarkable antibacterial properties against a variety of bacteria, which provided a strong experimental basis for the development of new antibacterial materials.

### 3.5. Comparisons of Performance with Other Pullulan-Based Films

Table 4 lists performance comparisons with other pullulan-based films. For an excellent packaging material, ideally, the mechanical strength, antioxidant (high DPPH and ABTS^+^) and antibacterial performance (higher antibacterial inhibition zone) should be as high as possible. At the same time, WVP should be as low as possible. By comparison, according to Table 4, the GPE film, especially GPE4, demonstrated lower WVP than many pullulan-based films reported. For example, pullulan/nano-TiO_2_’s performance in WVP was 1.8333 × 10^−10^·g.m^−1^ s^−1^·Pa^−1^, while the WVP of GPE4 was determined to be 0.7672 ± 0.2689 × 10^−10^·g.m^−1^ s^−1^·Pa^−1^. In cases where the reported WVP (for example, Adipic acid hydrazide-modified oxidized pullulan: Chitosan- 0.5556 × 10^−10^·g.m^−1^ s^−1^·Pa^−1^), the tensile strength (18 MPa) and the elongation break (4%) were reported to be significantly lower than GPE4 (tensile strength-35.08 ± 0.63 MPa, elongation at break-69.98 ± 1.58%). Similarly, the extract of pullulan/xanthan gum/grape seed from our previous work [68] also demonstrated lower WVP than our previous work but also lower mechanical strength than GPE4. Meanwhile, GPE4 also showed relatively stronger antioxidant performance or at least comparable performance to other reported works listed in Table 4. For example, the GPE4’s DPPH, which corresponds to the performance of anti-oxidant, was 76.64 ± 0.94%, which was significantly higher than most reported pullulan-based films, such as pullulan/xanthan gum/grape seed extract [69] and corn starch/pullulan/gallic acid. Furthermore, the antibacterial performance based on the antibacterial inhibition zone of GPE4 was also comparable to many pullulan-based films reported, such as hexamethylenediamine/pullulan/sodium alginate and diethylenetriamine/pullulan/sodium alginate. In cases where the antibacterial inhibition zone is much higher than GP4, such as oxidized pullulan modified with adipic acid hydrazide. The mechanical strength of chitosan, corn starch/pullulan/gallic acid and pullulan/xanthan gum/grape seed extract is lower than GPE4. Therefore, an excellent film based on pullulan, guar gum and LLE composite has been successfully developed with a good balance of low WVP and high mechanical strength and excellent antioxidant and antibacterial properties.

### 3.6. Food Packaging and Preservation Application Performance of the GPE Film

**Figure 6:**
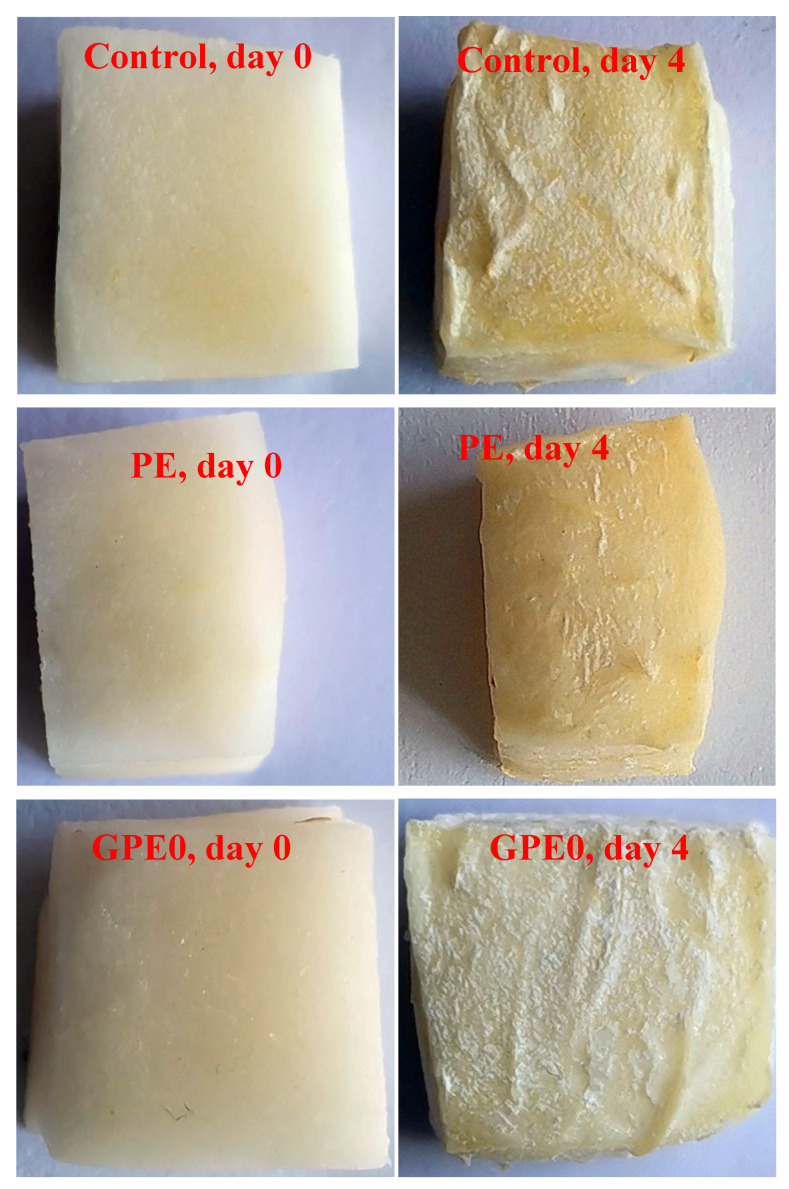

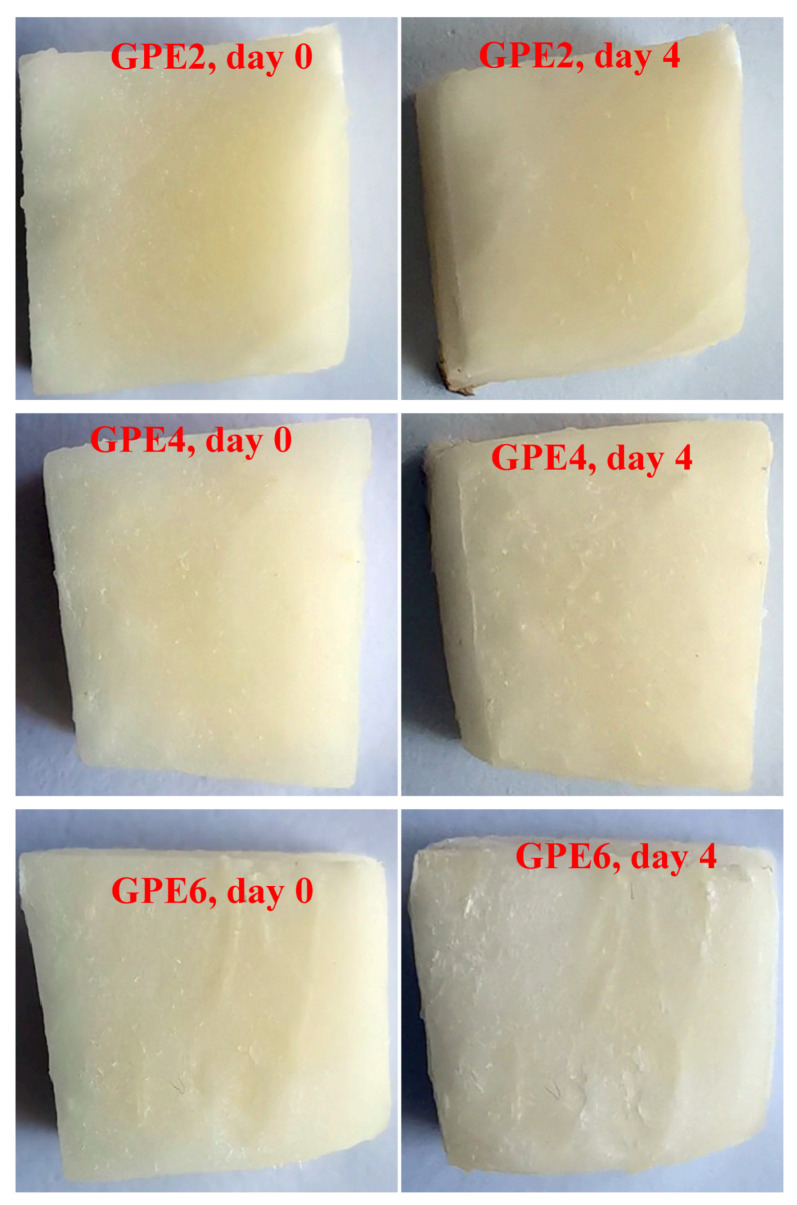
Preservation of CWC on day 0 and day 4.

In previous sections, it was found that LLE can effectively improve some physical properties of the composite film, but testing its preservation performance on fresh-cut fruits is still necessary. Therefore, the preservation performance of fresh-cut CWCs wrapped in four types of films (GPE0, GPE2, GPE4, and GPE6) developed in this study was investigated. Additionally, for comparisons, the preservation performance of fresh-cut CWCs without packing (control) and wrapped in conventional plastic (PE) films was also investigated. Simultaneously, the appearance and relevant indicators of fresh-cut CWCs were assessed to explore the potential of the composite film as food packaging. As shown in Figure 6, after 4 days of storage, the water loss of the CWC in the control sample was relatively severe, the surface was wrinkled and contracted, and the corners were raised. On the contrary, although there is slight water loss and wrinkles on the surface of pure PE film packaging samples, the corners are not tilted, and the water loss is not serious. However, significant color changes were observed in both unpacked CWC and in PE-packaged CWC, which became brownish after 4 days of storage. Meanwhile, some wrinkles also appeared on the surface of samples packed with GPE0, but to a lesser extent. However, the colors for all of the CWC packed with the GPE composite film remain the same as the fresh CWC, demonstrating that GPE composite film has great potential in commercial food preservation packaging. More importantly, no significant changes were observed in the GPE2, GPE4, and GPE6 samples after day 4. This suggests that the interaction between the LLE and guar gum/pullulan as a biodegradable film is important to ensure that CWC is preserved after 4 days. More importantly, it was much better than non-biodegradable PE film.

**Figure 7:**
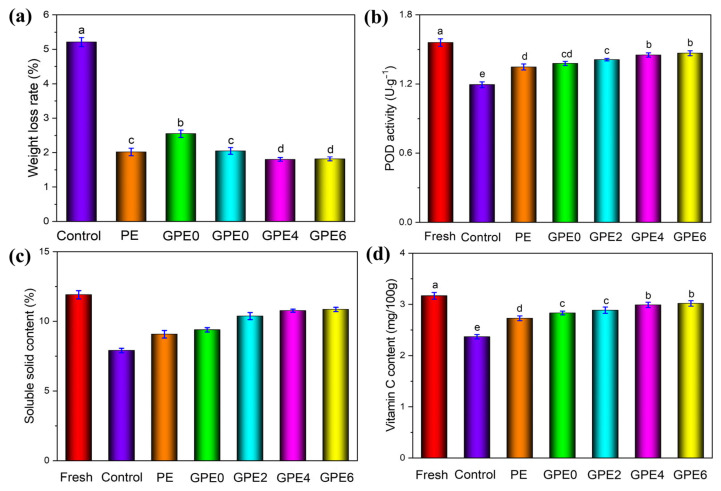
(**a**). Weight loss rate of fresh-cut CWCs with different packaging treatments at day 4; (**b**) Effect of different packaging treatments on POD activity of fresh-cut CWCs; (**c**) Soluble solid content of fresh-cut CWCs with different packaging treatments; (**d**) Vc content of fresh-cut CWCs with different packaging treatments.

With time, the amount of water in the CWC gradually decreases. Hence, the weight loss rate of fresh-cut fruit gradually increases, greatly affecting the quality of fresh-cut fruit. Therefore, controlling the fresh-cut fruit weight loss rate is essential to preserving food quality [69]. Figure 7a shows the weight loss rate of fresh-cut CWC with different packaging after storage for 4 days. The CWC in the control group was found to be directly exposed to the air due to the lack of any protective measures, and the mechanical damage resulting from its fresh cutting accelerated respiration, thus promoting substances’ weightlessness and consumption. Therefore, the weight loss of this group was as high as 5.21 ± 0.13%. On the contrary, the CWC in other packaging groups showed a better fresh-keeping effect, among which the weight loss of the GPE0 group was only 2.55 ± 0.11%. It is worth mentioning that when LLE was added to the composite film, its fresh-keeping effect could be further improved. Among the samples, the weight loss of the GPE4 sample was the smallest, which was only 1.80 ± 0.05%. This result is consistent with the lowest WVP value of the GPE4 film (Figure 7b), further proving the positive role of LLE in inhibiting water loss. At the same time, compared to the PE group (2.02 ± 0.11%), it was 10.89% lower. Therefore, biodegradable GPE4 film can solve the problem of water loss caused by transpiration and respiratory metabolism of fresh-cut CWC more effectively than non-biodegradable PE film. Meanwhile, there were no significant differences between the GPE4 film and the GPE6 film (*p* > 0.05). Therefore, 4% of LLE is the optimum amount.

Peroxidase (POD) helps maintain the quality and flavor of fruits and prolongs their fresh-keeping period by protecting the fruits against reactive oxygen species that cause oxidative damage to fruit tissue [70]. Figure 7b, effects of various packaging films on the activity of POD in fresh CWCs. The results show that the POD activity of fresh CWCs is the highest, but after four days of storage, the POD activity of CWCs in the control sample decreased significantly, from the initial 1.560 ± 0.033 U/g to 1.194 ± 0.025 U/g. This change is mainly due to the decline in CWC quality, tissue aging, and the slowing of intracellular metabolic activity during storage, leading to a decrease in enzyme activity. However, CWCs packed with PE and GPE films demonstrated excellent and comparable performance in maintaining POD activity. After storage for 4 days, the POD activity of the fresh-cut CWCs treated with PE remained at 1.348 ± 0.026 U/g, while the POD activity of the CWCs packaged with the GPE4 film containing LLE was as high as 1.452 ± 0.019 U/g. In particular, the POD activity of CWCs treated with GPE4 was 21.61% higher than that of the unpacked water chest and 5.29% higher than that of GPE0. The results showed that the LLE-containing film could significantly inhibit the decrease in the POD activity rate in fresh CWCs. Further analysis showed that the effects of the composite film with different LLE content on POD activity in fresh-cut CWCs were significantly different. Therefore, the composite film containing a proper amount of LLE can not only effectively inhibit the decline in POD activity in CWCs but also help maintain its freshness, thus significantly prolonging the fresh-cut CWCs.

The total amount of water-soluble compounds in food, which has a decisive influence on the taste, flavor, and nutritional value of fresh-cut fruit, is measured based on the soluble solid content. The effect of various packaging films on the change in the fresh-cut CWC’s soluble solid content during storage is displayed in Figure 7c where the solid soluble content of fresh-cut CWCs was kept at 11.92 ± 0.29% at the initial storage stage. However, after 4 days of storage, the content of soluble solids in both the control and packaging samples showed an obvious downward trend. This is because the natural respiration of fresh-cut fruits during storage will consume the organic matter in the fruits and produce carbon dioxide and water, leading to the consumption of some soluble solids and a decrease in the overall content. At the end of the storage period, the soluble solid content of fresh CWCs in the control sample decreased significantly to 7.91 ± 0.16%, which was considerably lower than that in the PE group and the GPE film (*p* < 0.05). However, it should be noted that the solid soluble content of CWC packaged with GPE4 remained at a high level of 10.77 ± 0.11%, compared to that of the control sample (7.91 ± 0.16%) and PE (9.08 ± 0.27%). The results showed that the GPE film containing LLE was excellent in inhibiting the respiration of fresh-cut CWCs and effectively slowing the decline in the soluble solid content. Therefore, the LLE in the GPE film contributed to preserving the taste, flavor, and nutritional value of the fresh-cut CWCs, exceeding the performance demonstrated by PE.

The content of vitamin C (Vc) in food, which is important for maintaining health, will inevitably decrease over time. Therefore, effective packaging is essential to maintain Vc in food. The effects of various packaging films on the content of Vc in fresh CWCs are displayed in Figure 7d. Fresh CWCs were originally rich in Vc, but after storage for 4 days, the Vc content of the CWC without packaging (control) decreased significantly from the initial 3.168 ± 0.066 mg/100 g to 2.368 ± 0.043 mg/100 g. However, the CWC-packed PE and GPE films showed a good fresh-keeping effect. After four days of storage, the content of Vc in PE-packed fresh-cut CWCs remained at 2.728 0.0447 mg/100 g, while GPE4-packed fresh-cut CWCs packed with PE remained at 2.728 ± 0.047 mg/100 g, while fresh-cut CWCs packed with GPE4 was higher, reaching 2.989 ± 0.051 mg/100 g. It should be mentioned that the Vc content of CWCs packed with the GPE4 film containing LLE is not only 26.22% higher than that of unpacked CWCs but also 5.58% higher than that of CWCs treated with GPE0. More importantly, it was also 9.57% higher than PE. This result fully shows that the LLE-containing film can effectively prevent Vc oxidation in fresh-cut CWCs, showing stronger antioxidant capacity than that of PE. Furthermore, the effects of the amount of LLE on the content of Vc in fresh CWCs were significantly different. Compared to GPE0 and GPE2, the retention effect of GPE4 on Vc was more significant (*p* < 0.05), but the difference between GPE4 and GPE6 is not significant (*p* > 0.05). Thus, the composite film incorporating LLE can not only effectively slow the loss of Vc in CWCs but also aid in preserving its freshness, significantly extending the shelf life and storage duration of fresh-cut CWCs.

## 4. Conclusions

The GPE composite film was fabricated by combining guar gum and pullulan with LLE. The results indicate that LLE was able to enhance the properties of the guar gum/pullulan composite film because of the hydrogen bond interaction between them. This causes the film to be compact, significantly increasing the mechanical strength while decreasing the solubility of water and WVP. It was found that 4% (GPE4) is the optimum LLE amount. At this amount, a balance of great mechanical strength and excellent antioxidant performance, while at the same time, lower WVP and greater mechanical strength with comparable antibacterial performance, can be achieved by many pullulan-based films. Additionally, based on food packaging and preservation investigation, it was found that GPE films, particularly GPE4, were able to preserve the quality of CWC fruit in terms of freshness, weight loss rate, Vc, and POD activity after 4 days of storage effectively. By comparison, it was much better, or at least comparable to that of non-biodegradable PE. Therefore, it can be concluded that GPE film holds significant promise as a sustainable alternative packaging material for preserving fruit-based foods like CWCs, potentially replacing PE in the future.

## Figures and Tables

**Table 1:** Physical Properties of Compound Film.

Films	Thickness (mm)	Moisture Content (MC) (%)	Water Solubility (WS) (%)
GPE0	0.150 ± 0.009 ^a^	22.94 ± 1.67 ^a^	60.89 ± 1.25 ^a^
GPE2	0.152 ± 0.005 ^a^	20.60 ± 2.09 ^ab^	44.25 ± 1.38 ^b^
GPE4	0.155 ± 0.007 ^a^	19.12 ± 0.77 ^b^	40.80 ± 0.84 ^c^
GPE6	0.157 ± 0.009 ^a^	18.62 ± 0.73 ^b^	39.03 ± 2.57 ^c^

**Table 2:** Tabulated data of light transmittance.

Sample	Light Transmittance (%)						
	250	300	350	380	400	500	800
GPE0	0	29.38	37.07	54.45	71.78	82.04	84.72
GPE2	0	0.058	33.27	49.66	65.77	75.34	77.27
GPE4	0	0.011	24.55	36.22	47.86	54.70	54.95
GPE6	0	0	13.12	22.08	31.99	38.91	41.31

**Table 3:** Tabulated data on antibacterial properties.

Films	Diameter of the Inhibition Zone (mm)		
	*Staphylococcus aureus* (ATCC6538)	*Escherichia coli* (ATCC25922)	*Bacillus subtilis* (ATCC6633)
GPE0	6.00 ± 0 ^d^	6.00 ± 0 ^c^	6.00 ± 0 ^d^
GPE2	7.82 ± 0.21 ^c^	8.77 ± 0.17 ^b^	7.12 ± 0.07 ^c^
GPE4	9.84 ± 0.07 ^b^	10.83 ± 0.11 ^a^	8.86 ± 0.10 ^b^
GPE6	10.07 ± 0.09 ^a^	10.96 ± 0.11 ^a^	9.04 ± 0.09 ^a^

**Table 4:** Comparisons of WVP, mechanical strength, antioxidant activity, and antibacterial activity with other various pullulan-based composite films.

Samples	WVP (×10^−10^·g.m^−1^ s^−1^·Pa^−1^)	Film Thickness (mm)	Tensile Strength (MPa)	Elongation at Break (%)	Antioxidant		Antibacterial Inhibition Zone (mm)			Ref.
					DPPH (%)	ABTS^+^ (%)	*Staphylococcus aureus*	*Escherichia coli*	*Bacillus subtilis*	
Hexamethylenediamine/pullulan/sodium alginate	0.694	0.013 ± 0.015	34	5	-	-	12.5 ± 0.12	11.3 ± 0.15	-	[11]
Diethylenetriamine/pullulan/sodium alginate	0.833	0.022 ± 0.011	30	5	-	-	11.5 ± 0.03	10.1 ± 0.14	-	[11]
Pullulan/nano-TiO_2_	1.8333	-	15.9	45	-	-	-	-	-	[12]
Whey protein isolate/pullulan film with nano-SiO_2_	1.11	-	3.51± 1.71	162.26± 27.53	-	-	-	-	-	[13]
Polyaldehyde pullulan pectinase	-	-	-	-	86	86	-	-	-	[14]
Gelatin pullulan dialdehyde	5.0556	-	15.4	421	-	-	-	-	-	[15]
Adipic acid hydrazide-modified oxidized pullan: chitosan	0.5556	0.013 ± 0.004	18	4	-	-	17	-	-	[16]
Corn starch/pullulan/gallic acid	1.3128	0.168 ± 0.001	15.17 ± 1.05	64.04 ± 1.48	58.16 ± 0.17	-	22.33 ± 0.66	22.67 ± 0.36	-	[17]
Pullulan/xanthan gum/grape seed extract	0.68 ± 0.07	0.207 ± 0.009	12.5	22	40	-	14.18 ± 0.38	9.21 ± 0.39	8.54 ± 0.59	[68]
**GPE0**	**0.87282 ± 0.2246**	**0.150 ± 0.009**	**24.9 ±0.85**	**84.60 ± 2.83**	**13.87** **±** **1.25**	**15.60** **±** **0.82**	**6.00 ± 0**	**6.00 ± 0**	**6.00 ± 0**	**This work**
**GPE4**	**0.7672 ± 0.2689**	**0.155 ± 0.007**	**35.08 ± 0.63**	**69.98 ± 1.58**	**76.64 ± 0.94**	**81.53 ± 0.84**	**9.84 ± 0.07**	**10.83 ± 0.11**	**8.86 ± 0.10**	**This work**

## Data Availability

The original contributions presented in the study are included in the article. Further inquiries can be directed to the corresponding author.
